# Effects of Concomitant Intra-Aortic Balloon Pump Usage and Different Cannulation Techniques on Venoarterial Extracorporeal Membrane Oxygenation Support in Terms of Organ Perfusion

**DOI:** 10.21470/1678-9741-2023-0241

**Published:** 2024-11-18

**Authors:** Mustafa Mert Özgür, Tanıl Özer, Mehmet Aksüt, Mehmet Dedemoğlu, Ekin Can Çelik, İbrahim Çağrı Kaya, Murat Bülent Rabuş

**Affiliations:** 1 Cardiovascular Surgery Department, Health Science University, Kosuyolu High Specialization Education and Research Hospital, İstanbul, Turkiye; 2 Pediatric Cardiac Surgery Department, Umraniye Training and Research Hospital, Istanbul, Turkiye; 3 Department of Cardiovascular Surgery, Antalya Education and Research Hospital, Antalya, Turkiye; 4 Cardiovascular Surgery Department, Eskisehir City Hospital, Eskisehir, Turkiye

**Keywords:** Extracorporeal Membrane Oxygenation, Cost-Benefit Analysis, Perfusion, Cardiovascular System, Catherization

## Abstract

**Introduction:**

Various cannulation strategies for venoarterial extracorporeal membrane oxygenation (V-A ECMO) support are currently in use according to the clinical urgency and experience of the rescuing team. Although central V-A ECMO is considered more effective than a peripheral approach, the superiority of one cannulation configuration instead of another remains a controversial subject. This study mainly aims to compare the contribution of V-A ECMO circulatory support modalities to patients’ improvement according to various cannulation site strategies and additional usage of intra-aortic balloon pump (IABP).

**Methods:**

The study design involved the categorization of all patients into two groups: isolated V-A ECMO support and V-A ECMO plus IABP support. Secondly, we divided the patients into four groups considering V-A ECMO cannulation sites, such as central (aorto-atrial), axillo-femoral, femoro-femoral, and jugulo-femoral. We analyzed the parameters regarding the outcome for each group.

**Results:**

When comparing cannulation sites in relation to laboratory parameters for assessing organ perfusion, no statistically significant differences were observed among the groups. We found no statistically significant result within the groups affecting organ perfusion. The complication rates were higher in patients with concomitant IABP support, but the difference was not statistically significant likewise.

**Conclusion:**

V-A ECMO provides effective perfusion, no matter which cannulation site is preferred during the decision-making process, and the utilization of IABP support has no additional contribution to the outcomes. We believe that the most suitable strategy should be a tailor-made decision according to the clinical status of patients, the pathology, urgency, and cost-effectiveness.

## INTRODUCTION

**Table:** 

Abbreviations, Acronyms & Symbols	
AMI	= Acute myocardial infarction
AST	= Aspartate aminotransferase
BMI	= Body mass index
CAD	= Coronary artery disease
CMP	= Cardiomyopathy
CTEPH	= Chronic thromboembolic pulmonary hypertension
ECMO	= Extracorporeal membrane oxygenation
EF	= Ejection fraction
IABP	= Intra-aortic balloon pump
IE	= Infective endocarditis
LV	= Left ventricular
V-A ECMO	= Venoarterial extracorporeal membrane oxygenation

Cardiogenic shock is a clinical condition characterized by systemic hypoperfusion resulting from progressive depression of myocardial function^[1]^. Venoarterial extracorporeal membrane oxygenation (V-A ECMO), globally recognized and reliable, serves as a circulatory support method to alleviate the burden on the heart while maintaining systemic blood flow. This extracorporeal life support option affords patients the necessary time to recovery or to either transplantation or a long-term implantable left ventricular assist device^[2]^.

The implantation of V-A ECMO primarily involves two methods: peripheral and central. While central applications entail mediastinal exploration, peripheral approaches are generally preferred to avoid the need for mediastinal opening. The advantages and disadvantages of this differentiation, which is based on the cannulation strategy, also vary^[3,4]^.

The main challenges affecting clinical improvement encompass surgical complexity, perfusion dynamics, and a diverse range of complications^[5]^. Given the wide range of etiologies, various clinical manifestations, and patient-specific factors like anatomical suitability, individuals eligible for ECMO therapy constitute a large and heterogeneous population. Consequently, conducting direct scientific comparisons of treatment strategies within ECMO implantation procedures may yield more precise results. This approach aids in facilitating evidence-based decision-making processes, especially in complex clinical scenarios^[6-8]^.

This study’s primary objective is to assess the effectiveness of V-A ECMO circulatory support, considering the combined use of intra-aortic balloon pump (IABP) and different cannulation site strategies. Additionally, comparison of different cannulation strategies is discussed in relation to current controversy.

## METHODS

The study design involved the categorization of all patients into two groups: Group I received solely V-A ECMO support, while Group II received additional IABP support along with ECMO. Furthermore, patients were further stratified into four subgroups based on the cannulation sites utilized during ECMO administration: Group A received central cannulation (venous from the right atrium and arterial from the aorta), Group B had femoral vein-axillary artery cannulation, Group C underwent femoral vein-femoral artery cannulation, and Group D received jugular vein-femoral artery cannulation. Analysis of examination results and complications was conducted with respect to these designated groups. Limb perfusion and associated complications were assessed through routine extremity examinations recorded in patient files. Target organs for perfusion evaluation included the liver, kidneys, and extremities, with special attention given to serum lactate values as crucial markers of organ perfusion. Unfortunately, cerebral perfusion assessment was not feasible due to the absence of routine NIRS measurements during follow-up. Additionally, most patients were under sedation, rendering routine neurological examination scores non-standardized. To maintain consistency and mitigate potential complications from extended hospitalization, study records were confined to the initial five days of ECMO support.

All participants in this study provided informed consent to share their data, with personal identification information excluded. The study protocol received approval from the Institutional Clinical Trial Review Board (2019.3/4-172), and the research was conducted in strict adherence to the principles outlined in the Declaration of Helsinki.

### Patients and Data Collection

This retrospective study utilized data from patients aged 18 to 80 years who received V-A ECMO support at our institute between January 2013 and January 2018. Inclusion criteria comprised individuals experiencing cardiogenic shock following acute myocardial infarction (AMI), cardiomyopathy, post-cardiotomy syndrome, or post-transplantation cardiac failure. Patients receiving additional V-V ECMO support, those with non-cardiogenic shock, and those initially supported with V-A ECMO in another institution but later transferred to our intensive care unit were excluded. Additionally, patients who succumbed on the procedural day were not included. Ultimately, a total of 210 patients met the specified criteria and were included in the study.

### Statistical Analyses

The statistical analyses were carried out utilizing IBM Corp. Released 2015, IBM SPSS Statistics for Windows, version 23, Armonk, NY: IBM Corp. Normality of the variables was assessed using the Kolmogorov-Smirnov and Shapiro-Wilk tests. Continuous variables were presented as mean ± standard deviation, while categorical variables were expressed as percentages.

## RESULTS

The study participants had a mean age of 53.4 ± 16.8 years (range: 18 to 79), with a male predominance of 61.9%. The average body mass index was 26.9 ± 3.2 kg/m^2^. Among the diagnoses, coronary artery disease accounted for 28.6%, followed by cardiomyopathies at 23.3%.

Post-cardiotomy syndrome was the most common indication for ECMO support, representing 67.6% of cases, and the femoral venous-femoral arterial approach was the most frequently employed cannulation site. Demographic characteristics for each group are summarized in Table 1. Notably, only the body mass index showed a significant difference, being higher in Group II (*P*=0.02). There was no statistically significant distinction between the groups in terms of ECMO requirement. However, when comparing cannulation sites, Group I exhibited a significantly higher rate of central cannulation, while Group II showed a markedly greater prevalence of femoral venous-axillary arterial cannulation (*P*=0.0016 and *P*=0.001, respectively). Operational variables specific to each group are also detailed in Table 1.

**Table 1:** Demographic features and operative data of the groups.

	Group I (ECMO)	Group II (ECMO + IABP)	*P*-value
Age (years)	54.2 ± 17.5	52.1 ± 16.1	0.39
Sex			0.48
Male	70 (59.8)	60 (64.5)	
Female	47 (40.2)	33 (35.5)	
BMI (Kg/m^2^)	26.4 ± 3.2	27.5 ± 3.2	0.02^*^
Primary diagnosis			0.25
CMP	23 (19.7)	26 (30.0)	
CAD	34 (29.1)	26 (28.0)	
CTEPH	19 (16.2)	5 (5.4)	
Aortic pathology	11 (9.4)	8 (8.6)	
Valvular pathology	14 (12.0)	12 (12.9)	
Combined cardiac pathology	15 (12.8)	14 (15.1)	
IE	1 (0.9)	2 (2.2)	
Preop. EF (%)	50 (35-60)	40 (25-55)	0.12
Cannulation site			
Central#	53 (45.3)	27 (29.0)	0.016^*^
Femoral vein-femoral artery	54 (46.2)	40 (43.0)	0.64
Femoral vein-axillary artery	9 (7.7)	22 (18.8)	0.001^*^
Jugular vein-femoral artery	1 (0.9)	4 (4.3)	0.1
Need for ECMO			0.31
Cardiomyopathy	22 (18.8)	22 (23.6)	
Acute myocardial infarction	6 (5.1)	6 (6.4)	
Post-cardiotomy syndrome	81 (69.2)	61 (65.6)	
Heart transplantation	8 (6.8)	4 (4.3)	

# Right atrial venous-aortic arterial cannulation

* Statistically significant parameter

BMI=body mass index; CAD=coronary artery disease; CMP=cardiomyopathy; CTEPH=chronic thromboembolic pulmonary hypertension; ECMO=extracorporeal membrane oxygenation; EF=ejection fraction; IABP=intra-aortic balloon pump; IE=infective endocarditis

Considering the observed complications following ECMO application, it is noteworthy that Group II exhibited a higher incidence of complications. Specifically, the requirement for dialysis was significantly elevated in Group II (*P*=0.002). Although the rates of peripheral ischemia and cerebrovascular events were higher in Group II, these differences did not reach statistical significance. A summary of complications by groups is provided in Table 2.

**Table 2:** Follow-up results and complications by groups.

Variables	Group I	Group II	Total	*P*-value
	(ECMO) (N: 117)	(ECMO + IABP) (N: 93)	N (%)	
	N (%)	N (%)		
Bleeding revision	45 (38.5)	46 (49.5)	91 (43.3)	0.11
Dialysis	14 (12.0)	27 (29.0)	41 (19.3)	0.002^*^
Cerebrovascular event	21 (17.9)	24 (25.8)	45 (21.4)	0.16
Peripheral ischemia	14 (12.0)	14 (15.1)	28 (13.3)	0.51
Pneumonia	18 (15.4)	15 (16.1)	33 (15.8)	0.88

* Statistically significant parameter

ECMO=extracorporeal membrane oxygenation; IABP=intra-aortic balloon pump

When comparing Group I (ECMO only) and Group II (ECMO + IABP) in terms of laboratory parameters and urine output, no significant differences were observed between the groups regarding indicators of liver function such as international normalized ratio (or INR), aspartate aminotransferase (AST), bilirubin, as well as parameters reflecting kidney function including urea and creatinine. Moreover, urine output was notably higher in Group II during the initial two days (*P*=0.01 - *P*=0.02). Although not statistically significant, the lactate value, recognized as a marker of organ perfusion, was found to be lower in Group I.

Patients were further categorized into four groups based on the cannulation sites employed during ECMO support: Group A (central, venous-aorta from the right atrium), Group B (femoral venous-axillary arterial), Group C (femoral venous-femoral arterial), and Group D (jugular venous-femoral arterial). Upon analyzing overall follow-up results and complications by cannulation sites, no significant differences were observed among the groups. Interestingly, when assessing dialysis requirements, it was noted that Group B exhibited a significantly higher need for dialysis (*P*<0.001). Although we anticipated disparities in terms of peripheral ischemia between the groups, no statistically significant differences were identified. Nonetheless, Group A, utilizing central cannulation, demonstrated the lowest incidence of peripheral artery ischemia. Furthermore, Group C displayed a notably higher frequency of pneumonia, reaching statistical significance (*P*<0.03) (Table 3).

**Table 3:** Follow-up results and complications by cannulation sites.

Variable	Group A	Group B	Group C	Group D	*P*-value
	(N: 80)	(N: 31)	(N: 94)	(N: 5)	
	N (%)	N (%)	N (%)	N (%)	
Bleeding	40 (50.0)	12 (38.7)	38 (40.4)	1 (20.0)	0.37
Dialysis	8 (10.0)	15 (48.4)^f^	18 (19.1)	0	< 0.001^*^
Cerebrovascular event	13 (16.2)	10 (32.3)	20 (21.3)	2 (40.0)	0.22
Peripheral ischemia	7 (8.8)	6 (19.4)	15 (16.0)	0	0.29
Pneumonia	9 (11.2)	2 (6.5)	22 (23.4)^f^	0	0.03^*^

* Statistically significant parameter

f Group that makes statistically significant difference

Group A: Central cannulation

Group B: Femoral venous-axillary arterial cannulation

Group C: Femoral venous-femoral arterial cannulation

Group D: Jugular venous-femoral arterial cannulation

When comparing cannulation sites in relation to laboratory parameters for assessing organ perfusion, no statistically significant differences were observed among the groups with regard to liver and kidney function, as well as the accepted marker of organ perfusion, lactate value. It's worth noting that five patients with femoral arterial-jugular venous cannulation in Group D were excluded from the analysis.

In the monitoring of ECMO support, the rates of “independence from peripheral artery ischemia” for groups with and without IABP are outlined in Table 4. Notably, the rate of independence from peripheral artery ischemia within the first five days exceeded 94% for all patients and groups. While there was no statistically significant distinction in independence rates from peripheral artery ischemia between the groups, it is noteworthy that Group II exhibited lower independence rates within the initial five days.

**Table 4:** Independence ratios from peripheral arterial ischemia.

Independence from peripheral ischemia (%)	Day 1	Day 3	Day 5	*P*-value
Group I (ECMO)	98.3	98.3	98.3	0.81
Group II (ECMO + IABP)	97.8	94.1	94.1	
				
Group A	98.8	97.2	97.2	0.75
Group B	93.5	85.8	85.8	
Group C	98.9	96.6	95.1	
Group D	-	-	-	

Group A: Central cannulation

Group B: Femoral venous-axillary arterial cannulation

Group C: Femoral venous-femoral arterial cannulation

Group D: Jugular venous-femoral arterial cannulation

ECMO=extracorporeal membrane oxygenation; IABP=intra-aortic balloon pump

Regarding the cannulation site, the rates of “independence from peripheral artery ischemia” within the initial five days were consistently > 85% (Table 4). Although no statistically significant difference was observed, it is worth noting that the lowest independence rate was associated with femoral vein-axillary artery cannulation.

## DISCUSSION

Based on the 2022 reports from the Extracorporeal Life Support Organization (or ELSO), ECMO support has been used in 172,835 patients worldwide, addressing various underlying causes. In the adult population, ECMO support was used in 38,610 patients primarily for cardiac-related causes with increasing trend in different medical centres^[9]^. Additionally, other circulatory support options have been considered according to the needs.

The combination of ECMO and IABP in managing cardiogenic shock is a widely employed approach in numerous medical centers. In the literature, the concurrent use of these two interventions in cardiogenic shock has generated controversy, with no definitive consensus reached. In a meta-analysis conducted by Vallabhajosyula et al.^[10]^, which examined 4,563 patients from 22 studies, the ECMO + IABP group was compared to the ECMO-only group. Interestingly, no significant difference in terms of survival was identified between the two groups.

In the meta-analysis conducted by Cheng et al.^[11]^, which encompassed 16 studies and 1,517 patients, it was reported that there was no discernible difference in terms of survival between the two groups. Furthermore, in the meta-analysis conducted by Huang et al.^[12]^, involving 2,251 patients, it was observed that there was no difference between the groups. Taken together, these studies highlight the ongoing debate regarding the potential benefits of incorporating IABP into ECMO application.

Most studies and meta-analyses in the literature have primarily assessed patients based on complications, such as survival, limb ischemia, and bleeding, when utilizing IABP and ECMO in tandem compared to using ECMO alone. Moreover, these studies often did not extensively consider variations in patient demographics and tended to focus on specific patient subsets. In terms of organ perfusion, while fewer studies exist, a series of 529 patients analyzed by Lin et al.^[13]^ compared similar groups for all cardiac causes. Parameters including urine output, lactate levels, and other markers of organ failure were evaluated, revealing no significant differences between the groups. When it comes to peripheral ischemia, it is noted that the use of IABP may lead to a higher incidence of fasciotomies, although no disparities were observed in terms of subsequent impairments. Furthermore, Bělohlávek et al.^[14]^ explored the effects of ECMO applications in various modalities following prolonged cardiac arrest in pigs. This study indicated that femoro-femoral V-A ECMO could provide adequate cerebral and myocardial perfusion, while the potential simultaneous use of IABP and ECMO might compromise coronary perfusion.

Left ventricular (LV) distension can occur in a range of 10% to 60% of patients receiving ECMO support. When used concurrently with V-A ECMO, the primary theoretical benefit of IABP lies in its ability to decompress the left ventricle, thereby reducing wall stress and enhancing coronary perfusion. However, it's important to note that IABP is not the sole option for achieving LV decompression. Techniques such as atrial septostomy, as well as direct or indirect LV venting, have demonstrated effectiveness in reducing cardiac pressures, pulmonary edema, and LV distension. Although these approaches appear effective in lowering LV pressure, none of them seems to show a significant impact on survival when used in conjunction with ECMO support^[15]^. A meta analysis by Fiorelli et al.^[16]^ reported a significant reduction in mortality rates with a combined use of Impella® device and ECMO support compared to ECMO alone during cardiogenic shock although at the expense of increased need for renal replacement therapy. On the other hand, a survival advantage of the Impella® device compared to IABP following AMI complicated by cardiogenic shock could not be confirmed by Alushi et al.^[17]^. Consequently, LV decompression may offer benefits primarily in specific cases. In our study, we observed that alongside ECMO support, the use of IABP may enhance organ perfusion, yet its definitive contribution to clinical improvement remains a topic of debate.

While IABP usage correlated with increased urine output, we also noted a higher frequency of dialysis requirement in patients with IABP support. At the conclusion of the five-day follow-up, there were no discernible differences between the groups in terms of liver function test results. However, it was observed that AST and total bilirubin values were higher in Group II (ECMO + IABP) on the first day, with no significant difference noted in the subsequent days. While the use of IABP resulted in an increase in urine output, it was noted that patients on IABP support showed a higher incidence of dialysis requirement. At the conclusion of the five-day follow-up period, no discernible differences were observed between the groups in terms of liver function test results. However, it was observed that on the first day, AST and total bilirubin values were higher in Group II (ECMO + IABP), with no notable differences on the subsequent day. The potential impact of IABP on liver enzyme elevation has been previously reported^[18]^. Furthermore, when examining serum lactate values - an important marker of organ perfusion linked to mortality in numerous studies - it is noteworthy that although higher lactate values were observed with the use of IABP, no statistically significant difference was found between the groups.

As previously mentioned, the choice of cannulation strategy depends on various factors including the specific disease, patient condition, and the urgency of the situation. Clinicians carefully weigh the pros and cons of different cannulation techniques when making decisions. The question of the ideal cannulation method for ECMO support, as well as which strategy (peripheral or central) offers superior perfusion and hemodynamic optimization, remains a subject of controversy. Unfortunately, the existing literature on this topic is somewhat limited. In a meta-analysis conducted by Raffa et al.^[19]^, encompassing 17 studies and 1,691 patients, it was reported that there was no significant difference in terms of survival, limb ischemia, cerebrovascular incidents, and sepsis between central, axillary, and femoral approaches. However, it was noted that the need for dialysis tends to be more common in cases of central cannulation.

In a study conducted by Saeed et al.^[20]^, the impact of cannulation technique on end-organ functions, hemodynamic parameters, and arterial blood gas values was compared between patients receiving femoro-femoral ECMO and central ECMO support. The analysis revealed that while there were not many statistically significant differences between the groups, bleeding complications were more frequently reported in cases of central cannulation. Similarly, in a study by Kanji et al.^[21]^ comparing femoro-femoral and central cannulations, no significant difference was observed in terms of mean peak lactate values - a marker of end-organ perfusion and peripheral ischemia. However, bleeding rates were found to be higher in cases of central cannulation.

Furthermore, in studies conducted by Wong et al.^[22]^ (103 cases) and Ranney et al.^[23]^ (131 cases), where axillary, femoral, and central cannulations were compared, no notable disparities were identified between the groups in terms of renal dysfunction, cerebrovascular accidents, and survival. Wong et al.^[22]^ noted a higher incidence of limb ischemia in femoral cannulation, while bleeding rates did not significantly differ between central and peripheral cannulation groups. Ranney et al.^[23]^ reported higher bleeding rates in central cannulation cases, and peripheral ischemia findings were more common in femoral cannulation.

In a study by Rastan et al.^[6]^ involving post-cardiotomy syndrome patients, 517 individuals were evaluated, with 60.8% receiving central cannulation, 11.8% axillary, and 27.4% femoral cannulation. The researchers reported that no clear superiority was observed among the cannulation methods. Throughout the study, due to complications in the central cannulation group, such as bleeding and infection, there was a shift towards peripheral cannulation. However, they encountered challenges in achieving adequate flow rates, leading them to revert to central cannulation. Additionally, they found that persistently high lactate levels were associated with low flow rates and increased mortality. The study also indicated similar mortality rates between cannulation strategies, but the percutaneous femoral vein approach was linked to decreased survival rates. In our study, no significant differences were observed between cannulation types in terms of kidney and liver function tests, as well as urine output. Similarly, there were no statistically significant disparities between the groups in terms of lactate values, which are considered important biomarkers of organ perfusion. Additionally, complications such as peripheral ischemia, cerebrovascular accidents, and bleeding did not significantly differ between the groups. However, it is worth noting that cases requiring revision due to bleeding were more prevalent in the central cannulation group.

Limb ischemia is considered a relatively common complication in ECMO patients, with reported rates of up to 34%, which may vary depending on the institution^[18]^. Among the various complications associated with ECMO, vascular complications, particularly in cases of femoral cannulation, are the most serious and impactful. In our study, femoral arterial cannulation was the predominant strategy, accounting for 44.8% of cases, and it was also the most commonly associated with limb ischemia. In this patient group, we observed a 95% freedom from limb ischemia during the five-day follow-up period. However, limb ischemia was still observed in 23% of the patients during the 30-day follow-up.

In theory, the axillary approach offers a retrograde flow pattern with less travelling route that closely mirrors the physiological flow achieved with central cannulation. Thanks to a well-developed collateral vascular network, the incidence of distal limb ischemia is notably lower compared to the femoral cannulation strategy. This complication becomes even rarer when utilizing grafts for axillary cannulation. However, it's important to consider the drawbacks of axillary cannulation, including the potential for hyperperfusion syndrome, increased procedural time, the introduction of a new surgical site, and the associated risks of bleeding and infection. Studies have reported that hyperperfusion syndrome occurs in approximately 15% of cases following axillary arterial cannulation, and in such instances, a fasciotomy may be necessary^[24-26]^. In our study, axillary artery cannulation was consistently performed using grafts. Notably, we did not encounter complications related to hyperperfusion syndrome or require intervention for limb ischemia.

## CONCLUSION

Our study showed that V-A ECMO could provide effective perfusion regardless of the cannulation site and that the use of IABP gave no additional contribution to the outcome. There is a need for the development of an effective approach to cardiogenic shock. Circulatory support with concomitant reduction of cardiac workload and potential complications may be achievable with a combination of currently available devices. Nevertheless, further studies need to provide the required evidence.
